# PKR downregulation prevents neurodegeneration and *β*-amyloid production in a thiamine-deficient model

**DOI:** 10.1038/cddis.2014.552

**Published:** 2015-01-15

**Authors:** F Mouton-Liger, A-S Rebillat, S Gourmaud, C Paquet, A Leguen, J Dumurgier, P Bernadelli, V Taupin, L Pradier, T Rooney, J Hugon

**Affiliations:** 1 Inserm UMR-S942, Paris 75010, France; 2 Department of Histology, Pathology and Biochemistry, Saint Louis Lariboisière Fernand Hospital, Service AP-HP, University of Paris Diderot, Paris, France; 3 Clinical and Research Memory Center, Paris Nord Ile de France Saint Louis Lariboisière Fernand Hospital, AP-HP, University of Paris Diderot, Paris, France; 4 Sanofi-Aventis Therapeutic Strategy Unit Aging, Chilly-Mazarin, France

## Abstract

Brain thiamine homeostasis has an important role in energy metabolism and displays reduced activity in Alzheimer's disease (AD). Thiamine deficiency (TD) induces regionally specific neuronal death in the animal and human brains associated with a mild chronic impairment of oxidative metabolism. These features make the TD model amenable to investigate the cellular mechanisms of neurodegeneration. Once activated by various cellular stresses, including oxidative stress, PKR acts as a pro-apoptotic kinase and negatively controls the protein translation leading to an increase of BACE1 translation. In this study, we used a mouse TD model to assess the involvement of PKR in neuronal death and the molecular mechanisms of AD. Our results showed that the TD model activates the PKR-eIF2*α* pathway, increases the BACE1 expression levels of A*β* in specific thalamus nuclei and induces motor deficits and neurodegeneration. These effects are reversed by PKR downregulation (using a specific inhibitor or in PKR knockout mice).

Thiamine (vitamin B1) deficiency (TD) induces regionally selective neurodegeneration in the mammal's brains, particularly in specific thalamus nuclei (submedial thalamus nuclei (SmTN) and ventral lateral nuclei (VLN)) and cerebellum.^1, 2^ TD-induced neuronal loss is associated with a chronic impairment of oxidative metabolism and neuroinflammation associated with glial activation.^3, 4, 5^ Diminished thiamine-dependent processes in humans is not only associated with Wernicke–Korsakoff syndrome but also accompany other neurodegenerative disorders, such as Alzheimer's disease (AD).^6^ Experimental TD has been used to model the pathogenesis and investigate the cellular mechanisms of neurodegenerative disorders. In mice, TD-induced oxidative stress enhances A*β* accumulation by regulating *β*-site APP-cleaving enzyme 1 (BACE1) maturation. These effects are amplified in AD mouse model.^7, 8^

The double-stranded RNA-dependent protein kinase (PKR) is one of the four mammalian serine–threonine kinases—the others being HRI (heme-regulated eukaryotic translation initiation factor-2*α* (eIF2*α*) kinase), GCN2 (general control nonderepressible 2) and PERK (protein kinase RNA-like endoplasmic reticulum kinase)—that catalyzes the phosphorylation of the *α* subunit of eIF2 in response to stress signals, leading to an inhibition of protein synthesis.^9, 10^ Activation of PKR is induced by various triggers such as viral infection and endoplasmic reticulum or oxidative stresses^11, 12^ and could be controlled by an interaction with its specific activator PACT (PKR activator), also named RAX in rodents. PKR phosphorylation is known to have a significant role in AD,^13^ and cerebrospinal fluid (CSF) PKR levels could be used as a potential diagnostic biomarker in AD patients.^14, 15^ PKR is a proapoptotic kinase^16^ and can exert a number of toxic effects on neurons that could contribute to the functional and pathological alterations in AD brains. PKR also contributes to neurodegeneration and to the pathological molecular mechanisms observed in AD. PKR can mediate Tau phosphorylation induced by A*β* exposure in cell cultures.^17^ Additionally, several investigators have demonstrated that eIF2*α* phosphorylation, via PKR-induced cellular stress, leads to increased BACE1 mRNA translation when, paradoxically, global protein translation is inhibited.^18, 19, 20, 21^ These alterations of BACE1 translational control could be explained by a stress-dependent phenomenon of translation initiation.^22, 23, 24^ Moreover, PKR and eIF2*α* are highly phosphorylated in SmTN and the cerebellum of TD mouse model. Analyses performed on cerebellar granule neurons exposed to a thiamine metabolic antagonist suggest that TD-induced neuronal death could be partially mediated by PKR activation.^25^

To date, all studies that have explored the deleterious role of PKR activation in neurodegeneration indicate that inhibition of PKR is a promising approach to inhibit pathological mechanisms. Moreover, recent studies have shown that the genetic lack of PKR enhances learning and memory in several behavioral tasks while increasing network excitability.^26^ The goal of this study was to assess the role of PKR downregulation on neurodegeneration and A*β* production in a mouse model of neurodegeneration.

## Results

### TD induces regional oxidative stress and inflammation

**Figure 1:**
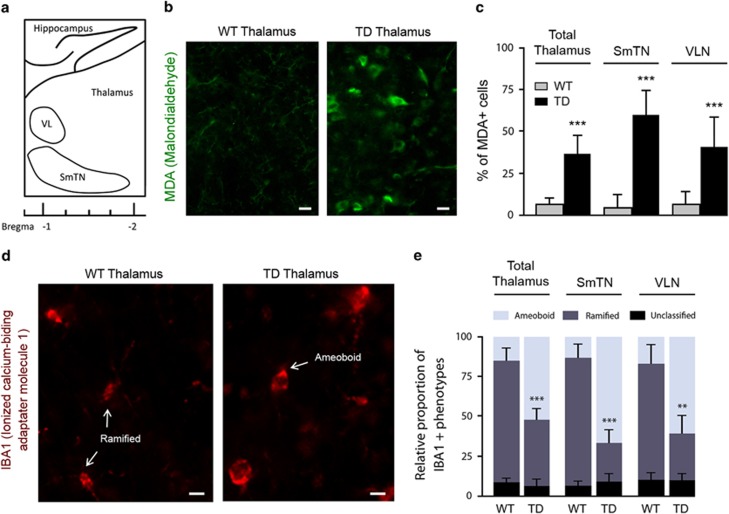
Markers of oxidative stress (MDA) and microglial cell activation (IBA1) in the TD thalamus. (**a**) Schematic sagittal representation of thalamic nuclei distribution: SmTN; and VLN. (**b**) MDA immunohistochemistry (green) on the sagittal sections of WT and TD thalamus. (**c**) Percentage of MDA-positive cells in total thalamus, SmTN and VLN in WT (*n*=6) and TD mice (*n*=6). (**d**) IBA1 labeling (red) on the sagittal sections of WT and TD thalamus. (**e**) Quantification of relative proportion of IBA1-positive microglial cell phenotypes: ramified shape (inactivated state) and amoeboid shape (activated state) in WT (*n*=7) and TD mice (*n*=7). Unclassified shape corresponds to intermediate state or cells that were unable to be quantified. Scale bars: 10 *μ*m. **P<0.01, ***P<0.001

To assess the consequences of the TD-induced stress cascade in our model, we evaluated the expression of markers of oxidative stress and microglial activation by immunofluorescence in the brain of mice exposed to 10 days of thiamine deprivation (TD mice) compared with wild-type mice (WT). Malondialdehyde (MDA) is the end product of lipid peroxidation and is commonly used as an oxidative stress marker. Using an MDA antibody, immunolabelings in TD mice brain showed a strong increase in staining in the cortical (not shown) and the thalamic neurons (37% of MDA-positive neurons). In the thalamus, this staining is mainly found in two well-defined thalamic regions (Figure 1a): the SmTN, which is located between bregma levels −0.94 and −1.94, and the VLN, between bregma levels −0.94 and −1.94.^27^ SmTN and VLN exhibit an increase of MDA-positive neurons, +57% and +42%, respectively (Figure 1c). Neurons in WT mice showed weak MDA staining (5.8% of MDA-positive neurons; Figures 1b and c). Microglia cells in their inactive state are characterized by a highly branched (ramified) morphology. After activation, they gradually undergo a transition from ramified to round feature (amoeboid). The ratio of these two phenotypical states both stained by an IBA1 antibody (ionized calcium-binding adaptor molecule 1) can be used as an indicator of microglial activation. Quantified immunofluorescence analyses of IBA1-positive microglia (IBA1+) in the thalamus revealed a 247% increase in the number of amoeboid cells in TD mice compared with controls (Figures 1d and e). The percentage of activated microglia was even greater in the SmTN (+343%) and in the VLN (+289%) (Figure 1d).

### TD enhances PKR activation in the thalamus and cerebellum

**Figure 2:**
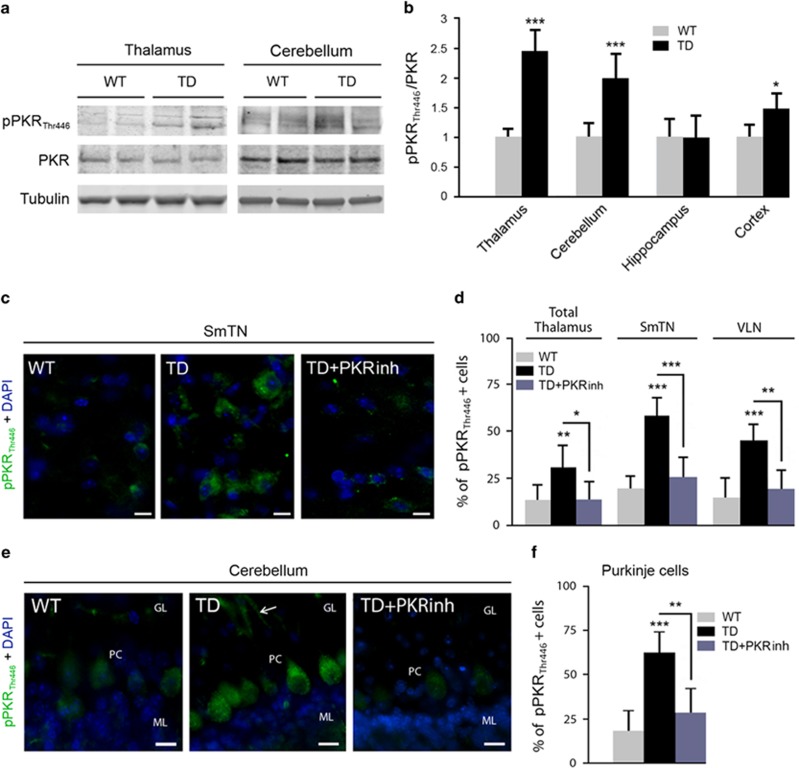
Activation of PKR in TD brain studied by immunoblotting and immunohistochemistry in the thalamus and cerebellum. Immunoblot analyses (**a**) and corresponding quantification of pPKR_Thr446_ and full PKR (**b**) in WT and TD mice protein samples, in the thalamus, cerebellum, hippocampus and cortex normalized on Tubulin levels. WT (*n*=14) and TD (*n*=14). *P<0.05, **P<0.01, ***P<0.001. (**c**) Immunohistochemistry (green) with a pPKR_Thr446_ antibody on WT, TD and TD pretreated with PKRinh mice in sagittal sections of (**c**) SmTN and (**e**) cerebellum. (**d**) The positive cell counting confirmed that PKR is activated after TD treatment and its increase is attenuated by PKRinh administration in total thalamus, specifically in SmTN and VLN. (**e**) Cerebellar pPKR_Thr446_ patterns in WT, TD and TD+PKRinh. pPKR_Thr446_ staining is almost absent in granular layer (GL) and molecular layer (ML) but exhibits strong signals and increased after TD in Purkinje cells (PC), both in cell bodies and processes (arrow). (**f**) The percentages of PC with pPKR_Thr446_ nuclear localization have been quantified, and as expected, pPKR_Thr446_ staining is decreased in PC after PKRinh administration WT (*n*=8) and TD (*n*=8). Scale bars: 10 *μ*m

To determine whether the inflammatory responses and oxidative stress affect the phosphorylation and expression of PKR in TD mice, immunoblotting with antibodies directed against the threonine (446) phosphorylation site or the total PKR protein were performed in the thalamus, cerebellum, cortex and hippocampus (Figure 2a). Quantification of the pPKR_Thr446_/PKR ratio showed a strong increase after TD in the thalamus, cerebellum and cortex of 139, 95 and 43%, respectively (Figure 2b). By contrast, there was no significant change in pPKR in the hippocampus (Figure 2b).

To explore the signaling pathway at the origin of PKR activation in TD, we performed immunoblots of the PKR main stress-dependent inducer, RAX, and the data revealed a significant increase in RAX in the thalamus and cerebellum (Supplementary Figure S1).

PKR knockout (PKR^−/−^) mice are viable, phenotypically indistinguishable from their wild type^28^ and have been used in several studies to test the protective role of PKR.^26, 29^ As expected, we did not detect a 65-kDa PKR band in brain samples of PKR^−/−^ mice (Supplementary Figure S2). We extended the above TD findings by counting pPKR_Thr446_-positive immunoreactive cells in the whole thalamus and in specific nuclei in the SmTN and VLN known to be more strongly affected by TD (Figures 2c and d) in animals pretreated (30 mg/kg per os) or not with a PKR inhibitor (PKRinh) provided by Sanofi. There was a significant enhancement of pPKR_Thr446_ expression after TD exposure. This effect was decreased after PKRinh pretreatment (−51% in total thalamus, −52% in SmTN, −54% in VLN) (Figure 2d) were observed in both structures. A comparable TD-induced increase of pPKR_Thr446_ staining was detected in the cerebellum where pPKR_Thr446_ is mostly present in Purkinje cell bodies and their arborizations (arrows) (+192%) (Figures 2e and f). In TD mice pretreated with PKRinh, the number of PKR-positive neurons was significantly reduced (58.9% Figure 2f).

### PKR inhibition reduces eIF2*α* activation in TD

**Figure 3:**
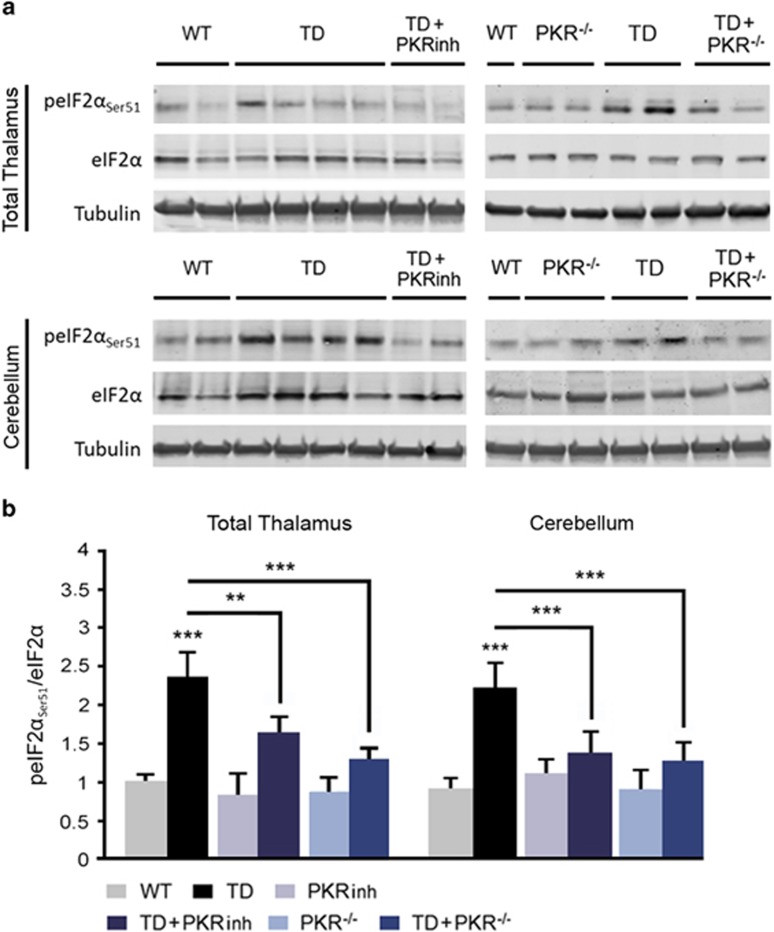
PKR inhibition controls eIF2*α* activity in TD mouse thalamic and cerebellar neurons. (**a** and **b**) Immunoblot analyses (**a**) and corresponding quantification (**b**) after normalization on Tubulin of peIF2*α*_Ser51_/eIF2*α* ratios in the three groups of mice non-exposed to TD (WT (*n*=14), PKRinh (*n*=10) and TD+PKR^−/−^ (*n*=12)) and in the three groups of vitamin B1-deficient mice (TD (*n*=14), TD+PKRinh (*n*=10) and TD+PKR^−/−^ (*n*=12)) in the total thalamus and cerebellum

**Figure 4:**
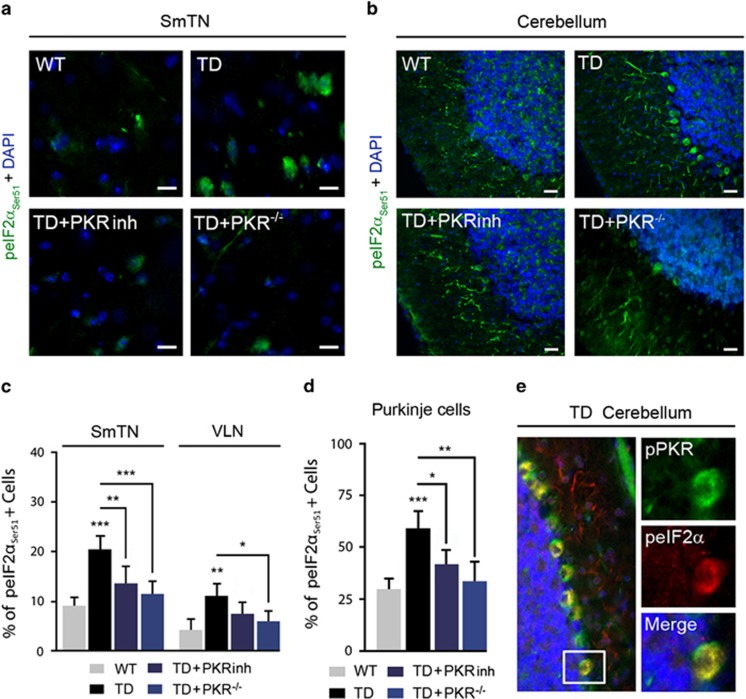
eIF2*α*_Ser51_ cellular localization in the thalamus and cerebellum in TD mice. (**a** and **b**) Double-labeling of DAPI (4,6-diamidino-2-phenylindole; blue) and peIF2*α*_Ser51_ (green) in the (**a**) SmTN and (**b**) cerebellum of WT, TD, TD+PKRinh and TD+PKR^−/−^ mice. peIF2*α*_Ser51_ in cerebellum is present in Purkinje cells' cytoplasm and mainly increase after TD at the level of the cells bodies. Scale bars: 10 *μ*m. (**c** and **d**) Quantification of positive neurons in SmTN and (**c**) VLN and (**d**) in Purkinje cells suggests that peIF2*α*_Ser51_ phosphorylation is dependent of pPKR_Thr446_ activation. *P<0.05, **P<0.01, ***P<0.001. (**e**) Confocal analysis showed a colocalization of pPKR_Thr446_ (green) and peIF2*α* (red) in TD cerebellum of the cerebellum. Boxes represent higher magnification at the level of Purkinje cells, where colocalization (yellow) is mainly present in cell bodies

To test whether TD-induced PKR activation could control eIF2*α* phosphorylation, we performed immunoblot analyses in mice pretreated or not with PKRinh or in PKR^−/−^ mice. PKRinh inhibits eIF2*α* phosphorylation in the thalamus (−31%) and cerebellum (−39%) (Figures 3a and b). After genetic knockdown of PKR, TD-eIF2*α* activation was also reduced (Figure 3b). Quantified immunofluorescence of peIF2*α*_Ser51_-positive neurons has confirmed these effects in the studied structures (Figures 4a and b). We also found that immunostaining for peIF2*α*_Ser51_ has a neuronal localization in the thalamus in the ventral posterolateral nucleus (VPL), with the strongest labeling in the SmTN and to a lesser extent in the VLN (Figure 4c).

In the cerebellum, TD-dependant activation of peIF2*α*_Ser51_ was predominantly observed in cell bodies and processes of Purkinje cells. As expected, peIF2*α*_Ser51_ staining is reduced by downregulation or inhibition of PKR (Figures 4b and d). The expression of peIF2*α*_Ser51_ signals showed a high degree of colocalization with pPKR_Thr446_ staining in the thalamus (not shown) and in cerebellar Purkinje cells (Figure 4e). An increased eIF2*α* phosphorylation was also detected by immunoblot or immunofluorescence in cortical structures (Supplementary Figures S3a and b) but not in the hippocampus (Supplementary Figure S3a). Of the four known eIF2*α* kinases, the most likely to be induced by vitamin B1 deficiency is PKR. Therefore we tried to determine whether PERK, activated by the ER stress/unfolded protein response, GCN2 and HRI could contribute to TD-eIF2*α* activation. Immunoblots of the thalamus, cerebellum and hippocampus samples from TD and control mice did not reveal any statistically significant variations of the pPERK_Thr980_/PERK ratio and HRI levels (Supplementary Figures S3c and d). However, we observed a 38.3% increase (*P*=0.044) of pGCN2_Thr899_/GCN2 ratio in the compared with control (Supplementary Figure S3e).

### PKR inhibition reduces neuronal loss and neurodegeneration owing to TD

**Figure 5:**
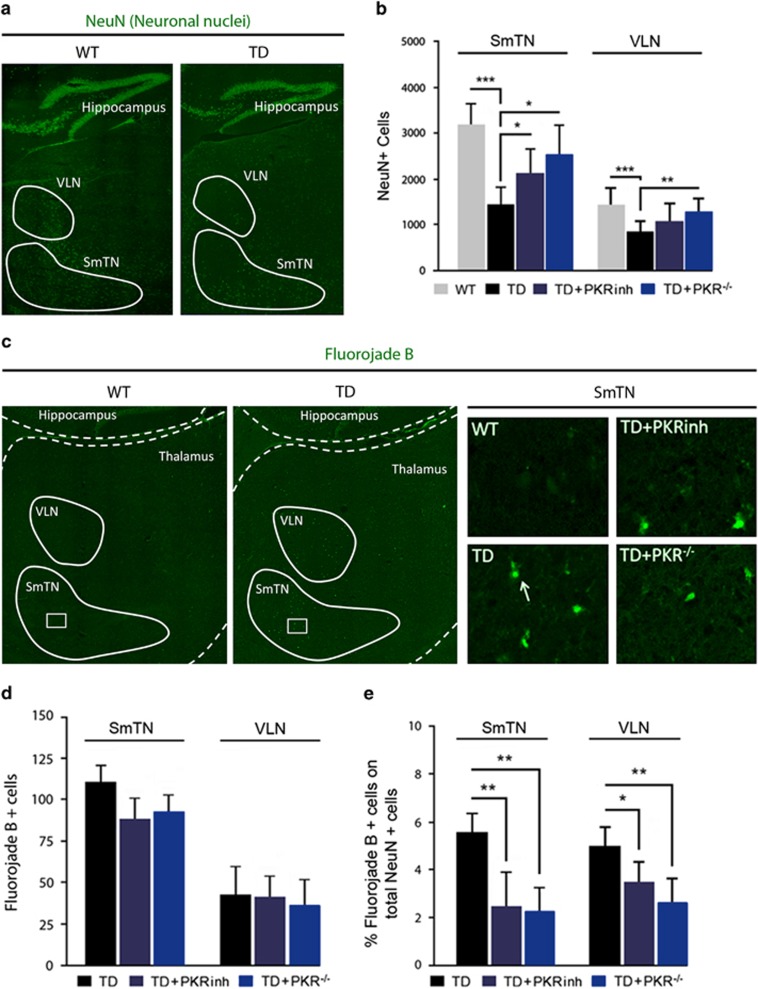
Regional TD-induced neuronal loss and neurodegeneration in the thalamus reduced by modulation of PKR activation. (**a**) Immunohistochemistry of neuronal nuclear marker NeuN (green) shows a decreased number of neuron in the TD thalamus compared with control mice, in particular in SmTN and VLN (surrounded areas). (**b**) The number of marked NeuN cells' evaluation indicates that TD-induced neuronal loss is reversed by PKR inhibition in the SmTN and in VLN. WT (*n*=8), TD (*n*=8) TD+PKRinh (*n*=8) and TD+PKR^−/−^ (*n*=7). (**c**) F-J B histofluorescence in thalamic regions (surrounded nuclei: SmTN and VLN) reveals, as visible in higher magnification boxes, neurodegenerative neurons in TD mice. Note that WT thalamus did not present a positive signal. (**d** and **e**) Relative analysis as number of F-J B+ cells in SmTN and VLN (**d**) or the percent normalized on the mean number of NeuN+ (**e**). The percentage of TD-damaged neurons is decreased by PKR inhibition (TD+PKRinh and TD+PKR^−/−^). WT (*n*=8), TD (*n*=8) TD+PKRinh (*n*=8) and TD+PKR^−/−^ (*n*=7); *P<0.05, **P<0.01, ***P<0.001

The neuronal nuclei (NeuN) antigen was used as a specific neuronal marker to quantify neuronal cell loss under various pathological conditions. Cell counting of NeuN-positive neurons in TD and control mice did not reveal any significant decrease in the various brain areas evaluated: cortex, cerebellum, hippocampus, and thalamus (data not shown). However, the evaluation in two specific thalamus nuclei (Figure 5a), SmTN and VLN, displayed a significant decrease of neuron density (54 and 37%, respectively) (Figure 5b). The pretreatment of mice with PKRinh before TD induction or TD context in knockdown PKR (PKR^−/−^) significantly reduced neuronal loss observed in the TD-SmTN (−73%). Similar protective effect have been found by pretreating TD mice with PKRinh in SmTN (−42%) and VLN (−39%). We then investigated the presence of damaged neurons in different thalamic areas using Fluoro-Jade B (F-J B), an anionic fluorescein derivative that has been reported to specifically stain degenerating neurons. As expected, we did not detect any staining in Wt mice (Figure 5c). In the TD thalamus, 97% of F-J B+cells were present in the SmTN or VLN. PKR inhibition did not affect the total number of F-J B-stained cells (Figure 5d). To take into account the decreased neuronal density owing to TD, we have adjusted the number of F-J B-positive neurons to the total number of neurons counted in Figure 5b. Downregulation of PKR significantly reduced the number of damaged neurons in TD SmTN (−62%) and VLN (−46%). Similar results were obtained in TD mice pretreated with PKRinh (57 and 30%, respectively) (Figure 5e).

On the contrary, we did not observe any effect of PKR inhibition on oxidative stress (Supplementary Figure S4) or microglial activation (Supplementary Figure S5). In summary, PKR inhibition prevents neuronal loss induced by TD but has no effect on TD-induced oxidative stress or neuroinflammation.

**Figure 6:**
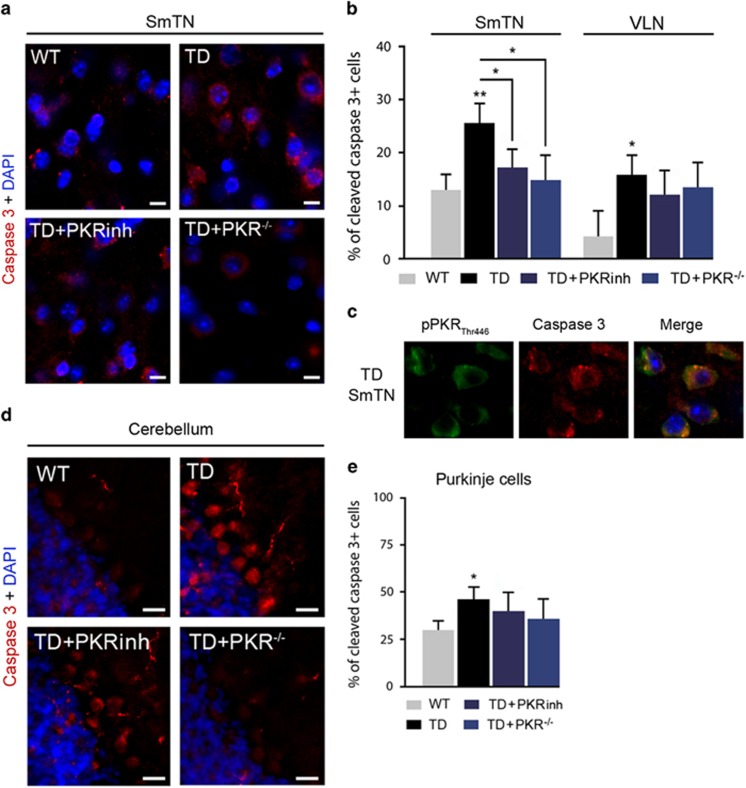
Immunolocalization of caspase 3 is enhanced in TD SmTN and Purkinje cells, and decreased by inhibition of PKR. (**a**) Double-labeling of nuclear marker DAPI (4,6-diamidino-2-phenylindole; blue) and caspase 3 (red) in SmTN of WT, TD, TD+PKRinh and TD+PKR^−/−^ mice. (**b**) The percentage of cleaved-caspase 3-positive neurons increased by TD is significantly reduced after downregulating PKR in SmTN (left) but not in VLN (right). (TD+PKRinh and TD+PKR^−/−^). (**c**) Confocal microscopy of double staining of caspase 3 (red)/PKR (green) performed with the Zenon Labeling Kit on coronal sections of TD submedial nuclei of the thalamus (SmTN) showing cytoplasmic colocalizations in neurons (yellow). (**d** and **e**) Caspase 3 immunolocalization in Purkinje cell layers (cell bodies and arborization) is amplified in TD models but is not reversed by downregulation of PKR. WT (*n*=8), TD (*n*=8) TD+PKRinh (*n*=8) and TD+PKR^−/−^ (*n*=7) *P<0.05, **P<0.01

**Figure 7:**
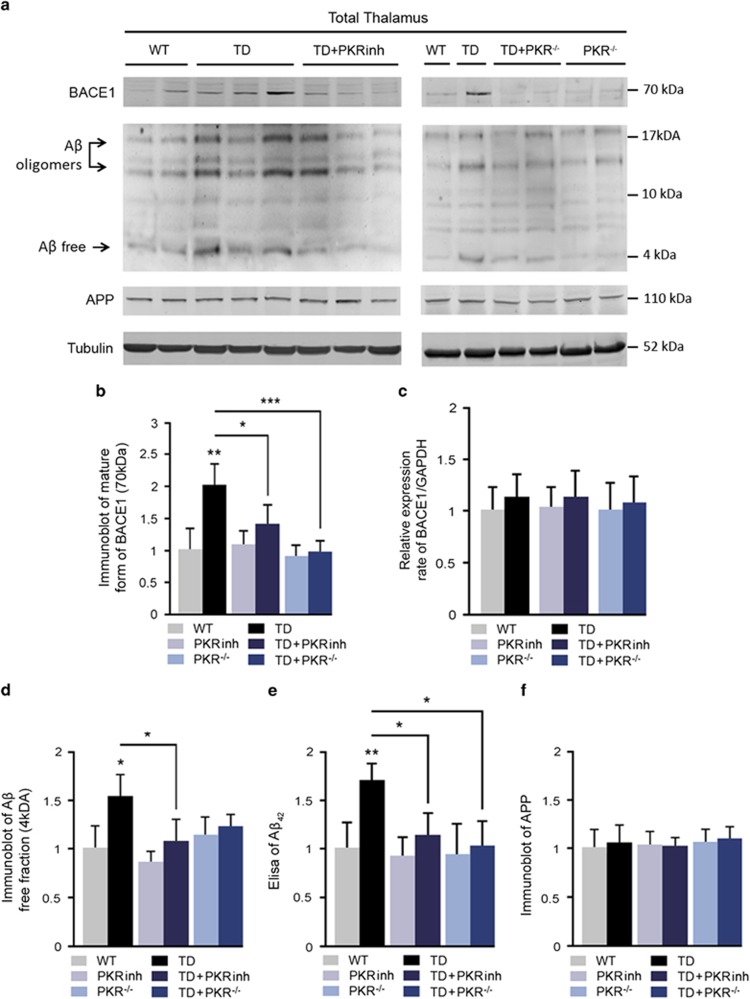
Inhibition of PKR-eIF2*α* pathway by PKRinh or in PKR^−/−^ mice decreases BACE1 levels and A*β* (Amyloid *β*) production. (**a**) Immunoblot on thalamus extracts and quantification of (**b**) BACE1, (**d**) A*β* oligomers and (**f**) APP. The blots show that TD induces BACE1 maturation and A*β* production without altering APP levels. PKR inhibition partially reduced the effects of TD. WT (*n*=8), PKRinh (*n*=8), PKR^−/−^ (*n*=7) TD (*n*=8), TD+PKRinh (*n*=8) and TD+PKR^−/−^ (*n*=7). (**c**) Transcriptional activity on BACE1 is not modified in the TD thalamus. Effect of TD on BACE1 mRNA levels is assessed with quantitative reverse transcriptase–PCR. BACE1 mRNA levels were normalized to GAPDH (glyceraldehyde 3-phosphate dehydrogenase) mRNA levels. WT (*n*=6), PKRinh (*n*=5), PKR^−/−^ (*n*=5) TD (*n*=6), TD+PKRinh (*n*=5) and TD+PKR^−/−^ (*n*=5). (**e**) The TD-dependent increase of A*β*_1–42_ peptide was confirmed by a sandwich enzyme-linked immunosorbent assay (ELISA) method and is expressed as means±S.E.M. A*β*_1–42_. The values from TD mice are expressed relative to the values from WT mice that were set to ‘1'. Immunoblots and ELISA were both performed on six different groups of mice: WT (*n*=8), PKRinh (*n*=8), PKR^−/−^ (*n*=7) TD (*n*=8), TD+PKRinh (*n*=8), and TD+PKR^−/−^ (*n*=7). *P<0.05, **P<0.01, ***P<0.001

Finally, to determine whether TD could lead to caspase 3-mediated apoptosis mainly in the SmTN, we quantified the number of neurons positive for a cleaved-caspase 3 antibody (Figure 6a). There was a large increase in apoptosis (+98% of positive cells) in the SmTN (Figure 6b). In addition, cleaved-caspase 3 stainings was also increased in TD Purkinje cells (+46% of positive cells) (Figures 7d and e). Interestingly, cleaved-caspase 3 and pPKR_Thr446_ staining showed a high degree of colocalization in the SmTN (Figure 6c) and cerebellum (not shown). Inhibition of PKR, either by PKRinh or in PKR^−/−^ mice, significantly reduced TD-induced caspase 3 levels in SmTN neurons (35 and 44%, respectively) (Figure 6b) but not in Purkinje cells (Figures 6d and e).

### PKR downregulation inhibited amyloidogenic amyloid precursor protein (APP) processing *in vivo* and A*β* production

Previous studies have shown that PKR-induced eIF2*α* phosphorylation could increase BACE1 translation.^18, 19^ To evaluate the functional importance of the PKR-eIF2*α* pathway in amyloidogenic signaling in TD mice, APP processing was assessed by immunoblotting of APP, BACE1 mature form and amyloid oligomers (Figure 7a). Quantified immunoblot demonstrated that BACE1 protein and amyloid *β* levels are significantly increased in the TD thalamus (by 110 and 52%, respectively) (Figures 7b and d). The TD-induced A*β* increase was confirmed by using ELISA assay for soluble A*β* (Figure 7e). By contrast, there were no changes in mRNA levels of BACE1 measured by real-time quantitative PCR (Figure 7c) or in APP protein expression in TD mice compared with control mice (Figure 7f). After 10 days of TD, PKR^−/−^ and PKRinh pretreated mice both exhibit a significantly reduced levels of A*β* levels in the thalamus (−39 and −33%). In summary, PKR blockade attenuates A*β* levels in TD mice.

We also investigated a potential role of PKR downregulation on Tau hyperphosphorylation by quantifying the levels of pTau on three hyperphosphorylated sites (AT8, AT100 and AT180) and of two kinases, JNK and GSK-3*β*, known to phosphorylate Tau in stress conditions. Comparisons of TD with control mice did not reveal any difference in AT100, AT180, GSK-3*β* and JNK activation ratio (Supplementary Figure S6).

### Decreasing PKR activation mitigates motor deficits

**Figure 8:**
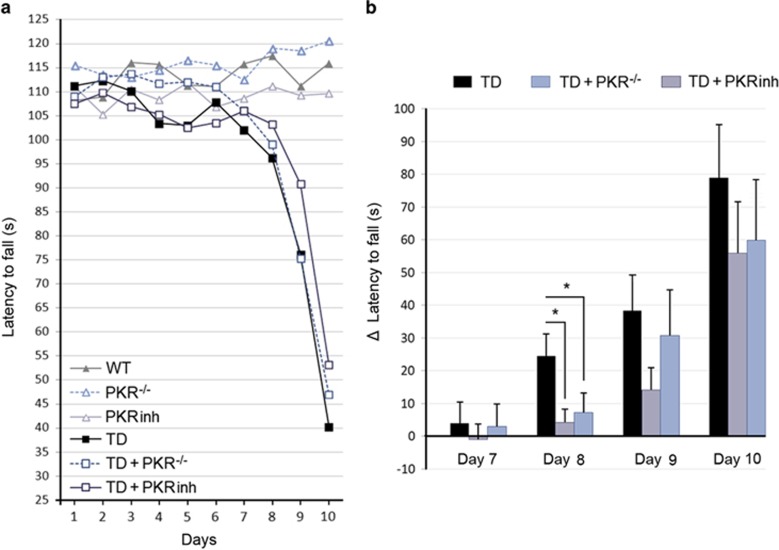
The rotarod motor performance impaired in TD mice is rescued after PKRinh administration or in PKR^−/−^ mice. (**a**) Diagram illustrating latency to fall measured on separate days reveals a strong decrease of motor performance in the three groups of TD mice. (**b**) Statistical analysis of measures for each animal of the delay between the rotarod performance between the first day of the experiment and on days 7, 8, 9 or 10. At the beginning of the decline (day 8), downregulation of PKR maintains motor performance of animals with TD. WT (*n*=14), PKRinh (*n*=14), PKR^−/−^ (*n*=12) TD (*n*=14), TD+PKRinh (*n*=14) and TD+PKR^−/−^ (*n*=12). *P<0.05

Marked motor deficits have already been described in several TD models.^30^ We therefore assessed motor coordination and balance in our TD mice using a rotarod apparatus (Figure 8a). Analyses of latency to fall in TD and control mice on an accelerating rotarod revealed a significant progressive decline of motor performance from day 8 in TD mice (Figure 8b). PKR downregulation, either by PKRinh administration or by using PKR^−/−^mice, significantly reduced motor decline at day 8 in TD mice (Figure 8b). After 10 days, motor performance scores of deficient mice were strongly impaired and were not modified by PKR inhibition. Rotarod results of TD mice have to be related to the general status of the mice, as evaluated in this study by body weight measurement (Supplementary Figure S7). In comparison to non-deficient mice, TD mice exhibit a dramatic weight loss starting at day 8 that is not reduced by PKR inhibition. Finally, we also measured pPKR_Thr446_ and peIF2*α*_Ser51_ in the gastrocnemius muscle in order to evaluate a role of PKR-eIF2*α* pathway inhibition in the muscle. Immunoblot analyses did not reveal any variation in the phosphorylation ratios of these two proteins (Supplementary Figure S8).

## Discussion

In the present study, we provide experimental data supporting the concept that ‘*in vivo*' inhibition of the double-stranded RNA activated protein kinase PKR, originally identified as a sensor of virus infection,^31^ has a neuroprotective action and can decrease A*β* production in a mouse model of neurodegeneration.

TD induces a strong increase of brain PKR activation, particularly in the thalamus and cerebellum. These observations are consistent with the previous study of Wang *et al.*,^25^ which showed a progressive increase of PKR phosphorylation on threonine 446 and 451, in these two structures, from the fifth day of TD. In our work, we have described the histological localization of pPKR_Thr446_ overexpression in TD brain. In the thalamus, TD-induced, PKR activation is mainly detected in the submedial and ventrolateral nuclei, which are also the brain areas most strongly affected by oxidative stress and inflammation. The expression of activated PKR in the cerebellum is mainly in the Purkinje layer that is partly comparable to the findings described by our group in the human brain affected by prion diseases that can trigger PKR activation.^32^ This expression pattern is also in line with the PKR mRNA profile visible on the Allen Brain Atlas, which shows a strong expression in Purkinje cells. Additionally, it is known that PACT, or its murine counterpart RAX, is responsible for cellular PKR activation in response to a variety of extracellular stresses.^33, 34^ After ethanol exposure, which shares common features with TD, RAX protein located in Purkinje layer interacts with PKR to reduce neuronal survival.^35^ After TD, we have also observed a significant increase of RAX levels in the cerebellum and at a lower magnitude in the thalamus, suggesting that RAX could mediate PKR activation in this TD cerebellum.

*In vivo* inhibition of PKR has been recently shown to exhibit significant positive effects such as enhancing memory and learning in behavioral tasks while increasing network excitability^26, 36, 37, 38^ and protecting animals from high-fat diet-induced insulin resistance and glucose intolerance.^29^ Previous studies in neuronal cultures have shown that PKR inhibition can attenuate neuronal death induced by various stresses, including A*β* neurotoxicity.^39, 40^ In the present study, we used a well-described PKR knockout model^28^ and a selective inhibitor of PKR activation (provided by Sanofi) to further evaluate the role of PKR in neurodegeneration *in vivo*. One of the major findings of this work is the validation that PKR inhibition can protect against neuronal loss in a model characterized by oxidative stress and neuroinflammation. Both stresses have been previously linked to PKR activation.^41, 42^ We demonstrated here that they act upstream of PKR-eIF2*α* pathway activation. However, these results did not allow us, at this stage, to determine the respective role in the TD model of neuroinflammation and oxidative stress in PKR activation.

Thalamic regional neuronal loss in TD rodents was described several times by the group of Gibson.^43, 44, 45^ Nonetheless, the cellular mechanisms underlying this neuronal loss are not fully understood. One of the major hypotheses to consider is the proapoptotic effect of PKR activation, through triggering of a downstream caspase cascade.^46^ By suppressing PKR in our TD model, we probably reduce neuronal loss due to apoptosis and, consequently, the proportion of damaged neurons. This possibility is consistent with the immunohistochemical results revealing that enhanced cleaved caspase 3 levels are strongly colocalized with pPKR_Thr446_ and significantly reduced after PKR downregulation in the thalamus. In the cerebellum, the increase of cleaved caspase 3 detected in Purkinje cells was not associated with a reduced number of these neurons. Ten days of TD in our model may not be sufficient to observe this phenomenon, which is detected in the humans brains affected by Wernicke's encephalopathy.^1^ Moreover, inhibition of PKR also leads to a decrease in the number of damaged neurons marked by F-J B staining in TD SmTN and VLN.

PKR can control the inflammasome and is involved in the general process of innate immunity and inflammation.^47^ However, we did not detect any modifications of the levels of microglial activation or oxidative stress in TD mice pretreated with PKRinh or in PKR^−/−^ mice. This findings suggest that, in the TD model, PKR inhibition acts downstream of the initial abnormal pathways associated with the lack of vitamin B1 and that the neuroprotective and functional effects obtained with PKR inhibition could be more related to the lack of activation of intrinsic or extrinsic apoptotic pathways^46^ or by a late regulation of autophagy.^48, 49^

During the past years, *in vitro* studies have demonstrated a role for eIF2*α* kinases and, in particular PKR, in the translational control of BACE1 levels and in the amyloidogenic pathway.^18, 19, 20^ Here we demonstrate a similar role for PKR on amyloid processes *in vivo*. The activation of eIF2*α* by TD is strongly colocalized with pPKR_Thr446_ and is reduced by PKR downregulation. In parallel, the levels of the other major stress-induced eIF2*α* kinases, activated PERK and HRI remain unchanged in the TD thalamus and TD cerebellum. Activated GCN2 is slightly increased only in the TD thalamus. Together the finding from PKR^−/−^ mice and the use of PKR inhibitor suggest that eIF2*α* phosphorylation is mainly under the control of PKR in this TD model. A previous report has revealed that TD increases *β*-secretase activity without effect on *γ*-secretase activity, which leads to an increase of A*β* production.^8^ Our results support this action on BACE1 levels and that PKR inhibition can attenuate this increase in amyloid production and BACE1 expression. A previous work has shown that OS can induce BACE1 transcription through JNK activation.^50^ The assessment of BACE1 mRNA levels and pJNK levels are not in favor of this possibility in TD model. Further data using JNK inhibitors in the TD model will be worth trying. Taken together, these results suggest that the increase of BACE1 levels in TD is regulated, at least in part, by a translational mechanism initiated by the PKR/eIF2*α* pathway. Zhao *et al.*,^51^ have demonstrated that pyrithiamine treatment and diet-induced TD exacerbated A*β* accumulation in the brain of APP/PS1 transgenic mice. On the other hand, they also described an enhanced number of phosphorylated Tau-positive cells associated with an alteration of GSK3-*β* activation. However, in our model, we have not observed any variations of Tau phosphorylation and GSK-3*β* activation, suggesting that an AD brain environment might be necessary for TD-dependent phosphorylation of Tau.

The major functional finding of our study is that using PKR^−/−^ or exposed TD mice to specific inhibitor PKRinh significantly reduced the decline of rotarod performance in TD mice at day 8. The use of the accelerating rotarod test, a highly reproducible method to assess the motor deficit in mice,^52^ was initiated according to the findings obtained in previous studies on TD models.^30^ In our model, a progressive performance decline was detected starting at day 7 in TD mice (Figure 7) and was transiently reduced at day 8 by PKR inhibition. This effect could be related to the neuroprotective effect of PKR inhibition in the thalamus and Purkinje cells and could be explained in several ways. (i) Neuronal loss occurs in the SmTN, which is a part of VPL and VLN of the thalamus. VLN serves as a central integrative functional division in the thalamic nuclei for motor control, receiving inputs from the cerebellum, striatum and cortex.^53^ (ii) Biochemical abnormalities of the PKR, eIF2*α* and caspase 3 in Purkinje cells, which are known to regulate and coordinate motor movements. Further electrophysiological studies analyzing Purkinje cells in TD model will be needed in the future. The strong and global imbalance of the physiological state of TD animals, as illustrated by the dramatic weight loss observed in all TD mice (with or without PKR suppression) could explain this lack of motor effect of PKR downregulation at days 9 and 10 of TD. Other motor performance tests carried out at the beginning of the decline could strengthen this hypothesis. Moreover, we cannot exclude that a part of the progressive motor impairment observed in our model could be due to metabolic changes induced by TD, in particular heart damage.

In conclusion, our results demonstrate that *in vivo* inhibition of PKR can allow neuroprotection and functional improvement in a TD model of neurodegeneration. In addition, PKR can modulate the *in vivo* production of A*β*. It is known that PKR is activated in the AD brains and other human neurodegenerative disorders^32, 54^ and that activated PKR is increased in the CSF of AD patients.^15^ In the future, pharmacological inhibition of PKR might therefore represent a valid target to slow neurodegeneration in several human diseases.

## Materials and Methods

### Mice

All animal experiments in this study were performed in accordance with the guidelines of the French Agriculture and Forestry Ministry for handling animals (decree 87849, license A75-05-22). PKR knockout (PKR^−/−^) mice were obtained by disruption of the Pkr gene introduced in embryonic stem cells, which were injected into C57BL/6 blastocysts. The chimeric mice were bred to C57BL/6 mice and the progeny homozygous for the disrupted allele, as previously published.^28^

TD was induced in 3-month-old C57BL/6 WT and PKR^−/−^ mice, following a previously described protocol.^43, 45^ Briefly, mice were housed in a controlled environment (one mouse/cage at 23 °C and 53% humidity). The animals were fed for 10 days with either a control diet (Safe, Augy, France) or a thiamine-deficient diet (Safe) *ad libitum*. TD animals additionally received a daily intraperitoneal injection of a thiamine pyrophosphokinase inhibitor,^55^ pyrithiamine hydrobromide (Sigma, St. Louis, MO, USA; 0.5 mg/kg body weight) while littermate control animals were injected with saline. Body weights were measured one time per day from days 0 to 10.

For brain preparation, mice were anaesthetized with pentobarbital (40 mg/kg, i.p.) and received an intracardial perfusion with cold PBS. Brains were removed and dissected on ice and then placed in 4% (w/v) paraformaldehyde in PBS for immunohistochemistry or immediately frozen in liquid nitrogen for immunoblotting or quantitative RT-PCR.

### Pharmacological inhibition of PKR

An inhibitor of PKR activation, with IC50 value of 2 nM (Reaction Biology Corporation, Malvern, PA, USA) was provided by Sanofi (Chilly-Mazarin, France) and administered daily by oral gavage (30 mg/kg). The IC50 values for the other eIF2*α* kinases are 14 nM for GCN2, 1030 nM for PERK and 1410 nM for HRI. The first administration of PKR inhibitor started one day before the beginning of the TD diet.

### Rotarod performance

The rotarod apparatus (Columbus Instruments, Columbus, OH, USA) was used to evaluate the motor coordination and balance of rodents and is especially sensitive in detecting cerebellar dysfunction. Mice were trained for 2 consecutive days to become acquainted with the rotarod apparatus. Motor performances were assessed in WT and PKR^−/−^ mice from days 1 to 10, with an acceleration range of 4–40 r.p.m. in 5 min. The latency to fall off the rotating rod per rotation speed level was recorded as a measure of motor function.

### Immunoblot analyses and ELISA

Thalamus, cerebellum, cortices, hippocampi and gastrocnemius muscles were homogenized and sonicated in a radio immune precipitation assay buffer containing 25 mM *β*-glycerophosphate, 50 mM sodium fluoride, 2 mM sodium pyrophosphate, protease inhibitor cocktail (Roche, Penzberg, Germany), 1 mM Sodium orthovanadate and 0.1 mM calyculin (Sigma) as phosphatase inhibitors. They were centrifuged at 20 000 × *g* for 10 min. The protein concentration was determined with the Micro BCA Protein Assay Reagent Kit (Thermo scientific, Cergy-Pontoise, France) using the manufacturer's protocol. Protein samples (25–40 *μ*g for each samples) were separated on gradient NuPAGE Bis-Tris gels (Invitrogen, Carlsbad, CA, USA) and then electroblotted onto nitrocellulose membranes (GE Healthcare, Chalfont St. Giles, UK) in 25 mM Tris, (pH 8.3), 200 mM glycine and 20% ethanol. After protein transfer, the membranes were blocked in 5% milk in TBS and then incubated with primary antibody.

The following primary antibodies were used in immunoblots: mouse anti-Amyloid *β* (Millipore, Billerica, MA, USA), rabbit anti-BACE1 (Santa Cruz, Danvers, MA, USA), mouse anti-phosphorylated GSK-3*β*_Tyr216_ (pGSK-3*β*_Tyr216_) (BD Transduction Laboratories, San Jose, CA, USA), rabbit anti-pGSK-3*β*_Ser9_ (Cell Signaling, Beverly, MA, USA), mouse anti-GSK-3*β* (Santa Cruz), anti-phosphorylated JNK_Thr183/Tyr185_ (pJNK_Thr183/Tyr185_), mouse anti-JNK (Santa Cruz), rabbit anti-phosphorylated PKR_Thr446_ (pPKR_Thr446_) (Santa Cruz), rabbit anti-PKR (Santa Cruz), goat anti-PACT/RAX (Santa Cruz), rabbit anti-phosphorylated PERK_Thr981_ (pPERK_Thr981_) (Santa Cruz), rabbit anti-PERK (Santa Cruz), rabbit anti-phosphorylated eIF2α_Ser51_ (peIF2α_Ser51_) (Cell Signaling), rabbit anti-eIF2α (Cell Signaling), rabbit anti-phosphorylated GCN2_Thr899_ (pGCN2_Thr899_) (Abcam, Cambridge, UK), rabbit anti-GCN2 (Cell Signaling), rabbit anti-HRI (Abcam), mouse AT8 (Ser202) (Innogenetics, Ghent, Belgium), mouse AT100 (Ser212/Thr214) (Thermo Scientific, Cergy Pontoise, France), mouse AT180 (Thr231/Ser235) (Thermo Scientific), mouse anti-Tau (Thermo Scientific), and mouse anti-Tubulin (Santa Cruz).

IR Dye 700DX conjugated anti-mouse IgG, IR Dye 800CW conjugated anti-goat IgG and IR Dye 800CW conjugated anti-rabbit IgG (Rockland Immunochemical Inc., Gilbertsville, PA, USA) were used as secondary antibodies. Bound proteins were visualized with the Odyssey Imaging System (Li-Cor Biosciences, Lincoln, NE, USA) and quantified with the Multigauge software (Fuji Film, Tokyo, Japan). ELISA was used to assess the A*β*_1–42_ levels in thalamus samples. Frozen extracts were centrifuged, and the soluble supernatant fractions were collected. The insoluble materials were then dissolved with lysis buffer containing 8% SDS, urea and 5 mM EDTA. A*β* levels were quantified using the mouse A*β*_1–42_ ELISA Kit (Invitrogen) following the manufacturer's instructions. The absorbance was read at 450 nm using a 96-well plate reader, and A*β* levels were calculated from a standard curve.

### F-J B histofluorescence

F-J B (a high-affinity fluorescent marker for the localization of neuronal degeneration) (Millipore) staining was carried out according to the method described in a previous study.^56^ In brief, the sections were first immersed in a solution containing 1% sodium hydroxide in 80% alcohol. They were then incubated in a solution of 0.06% potassium permanganate and transferred to a 0.0004% F-J B staining solution. With this method, neurons that undergo degeneration brightly fluoresce in comparison to the background.

### Immunohistofluorescence and confocal imaging

The other hemi-brain from each mouse was sagitally sectioned on a cryostat apparatus at 10 *μ*m. Sections were processed for immunohistofluorescence; blocking solution was PBS (phosphate buffered saline)/0.25% gelatin/5% normal donkey serum/0.3% Triton. Primary antibodies and secondary donkey antibodies were incubated at 4 °C for 24 h and at RT for 2 h, respectively, in PBS/0.125% gelatin/0.3% Triton. The primary antibodies used for immunofluorescence were as follows: rabbit anti-pPKR_Thr446_ (Sigma), rabbit anti-peIF2α_Ser51_ (Cell Signaling), rabbit anti-caspase 3 active fragment (BD Bioscience, San Jose, CA, USA), mouse anti-NeuN (Millipore), rabbit anti-MDA (Abcam), and rabbit anti-ionized binding molecule adaptor 1 (Iba1) (Wako, Osaka, Japan). Secondary antibodies were donkey anti-mouse Alexa Fluor 488 (Invitrogen) or donkey anti-rabbit Cy3 (Jackson Laboratory, Bar Harbor, ME, USA). For double confocal immunohistochemistry with antibodies from the same species, we used labeled Fab techniques. This approach need to label individually with the Zenon Kit (Sigma) one of the primary antibodies with fluorescent labeled-Fab, according to manufacturer's protocol.

Standard epifluorescence images were acquired on a Leica DMRD microscope using a high-resolution camera (Coolsnap HQ, Roper Scientific, Sarasota, FL, USA). Confocal imaging was performed using a Leica SP2 confocal microscope (Leica Microsystems, Solms, Germany). The Metamorph software (Roper Scientific) was used for image acquisition.

Counting of all the positive cells for pPKR, peIF2*α*, caspase 3, MDA, F-L B and NeuN were performed with the NIH ImageJ software (NIH Image, Bethesda, MD, USA), as previously described.^57^ DAPI and the cyanine 3 or alexa 488 pictures were both background corrected using the rolling ball method. A threshold was then chosen using the ‘Auto threshold' function of ImageJ. The distinct phenotypes of IBA1-positive microglial cells were blindly evaluated by manual counting and classified into three groups: ‘amoeboid', ‘ramified', and ‘intermediate' (also referred to as ‘unclassified').

### Quantitative RT-PCR

Total RNA was isolated using the TRIzol reagent (Invitrogen) from SH-SY5Y cells. SuperScript II Reverse Transcriptase Kit (Invitrogen) with random decamer priming was used to synthesize the first-strand cDNA from samples with an equal amount of total RNA (1 *μ*g), according to the manufacturer's instructions. cDNA samples were forwarded to amplification with specific primers for BACE1 and GAPDH using SYBR Green Technology (Invitrogen) and the Stratagene Mx3005P PCR cycler (Agilent Technologies, Massy, France). Human BACE1 primers (forward 5′-GCAGGGCTACTACGTGGAGA-3′ and reverse 5′-GTATCCACCAGGATGTTGAGC-3′), human GAPDH primers, (forward 5′-AATCCCA;TCACCATCTTCC-3′ and reverse 5′-GGACTCCACGACGTACTCA-3′) were used in the present studies. cDNA amplification was carried out as follows: denaturation at 94 °C for 2 min, followed by 35 cycles of denaturation at 94 °C for 15 s, primer annealing at 62 °C for 30 s, and extension at 72 °C for 40 s. A final extension was carried out at 72 °C for 10 min, ending with a 4 °C hold cycle. All assays were performed in triplicate, and BACE1 and PKR mRNA levels were normalized with the expression levels of GAPDH. Relative levels and gene copy numbers were calculated using the deltaCp method, as described by Pfaffl.^58^

### Statistical analysis

Statistical analysis was performed using GraphPad Prism 5 (GraphPad Software, Inc., La Jolla, CA, USA). Results were considered significant when *P*<0.05 using a non-parametric Mann and Whitney test.

## Abbreviations

AD: Alzheimer's disease
TD: thiamine deficiency
CSF: cerebrospinal fluid
SmTN: submedial thalamus nuclei
VLN: ventral lateral nuclei
BACE1: *β*-site APP-cleaving enzyme 1
PKR: double-stranded RNA-dependent protein kinase
PACT: PKR activator
HRI: heme-regulated eIF2*α* kinase
GCN2: general control nonderepressible 2
PERK: protein kinase RNA-like endoplasmic reticulum kinase
eIF2*α*: eukaryotic translation initiation factor-2*α*
MDA: malondialdehyde
NeuN: neuronal nuclei
F-J B: Fluoro-Jade B
IBA1: ionized calcium-binding adaptor molecule 1
APP: amyloid precursor protein
