# Supplementary material for: PKR downregulation prevents neurodegeneration and β-amyloid production in a thiamine-deficient model
Source: Cell Death Dis. 2015 Jan 15;6(1):e1594–. doi: 10.1038/cddis.2014.552 (PMC4669750; doi:10.1038/cddis.2014.552)
Supplement: Supplementary Figures [file cddis2014552x1.doc]

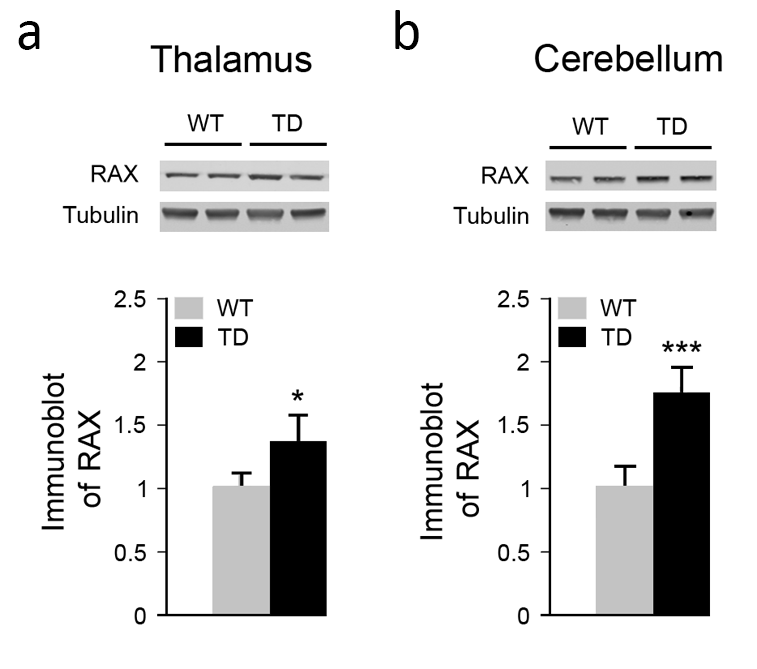


Supplementary Figure 1. Western blot experiments showing an increase of RAX, a cellular activator for double-stranded RNA-dependent protein kinase, both in TD thalamus (**a**) and cerebellum compared to controls (WT) (**b**). WT [n=14], TD [n=14]. * p<0.05, ***p<0.001.


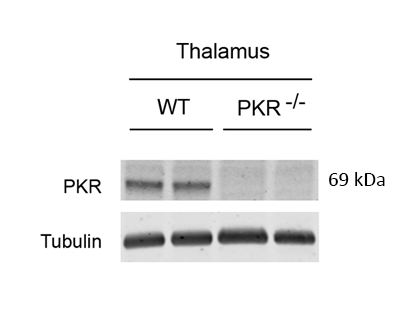


Supplementary Figure 2. Immunoblot performed on WT and PKR^-/-^ mice with N-terminal PKR antibody which as expected did not detect a 69-kDa PKR band in knock-out mice.


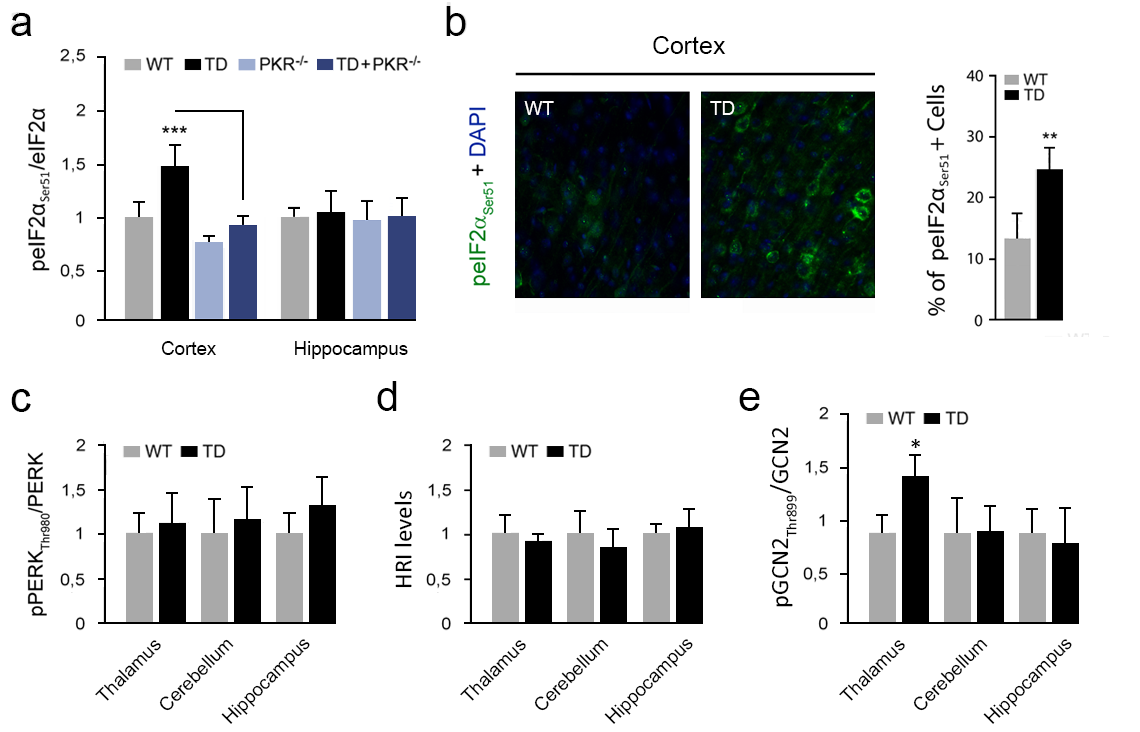


Supplementary Figure 3. eIF2α activation and eIF2α kinases level in TD brain structures.

(**a**) peIF2α_Ser51_/eIF2α ratio measured by immunoblot in hippocampus of WT, TD, PKR^-/-^ and in PKR^-/-^ with thiamine deficiency (TD+PKR^-/-^). **(b)** Immunofluorescence staining of peIF2α_Ser51_ showing a strong increase in TD cortical neurons compared to WT. ImageJ evaluation confirmed this observation by a significant increase of peIF2α_Ser51_ positive cells . PERK (PKR-like ER kinase) and pPERK_Thr980_ (**c**), HRI (**d**) and GCN2 and pGCN2_Thr899_ levels (**e**) were measured by western blot followed by statistical analysis in the following groups: WT and TD. pPERK_Thr980_/PERK ratios and HRI levels are unchanged after TD diet in  thalamus, cerebellum and hippocampus. pGCN2_Thr899_/GCN2 ratios are slightly increased only in the thalamus. WT [n=6], TD [n=6].


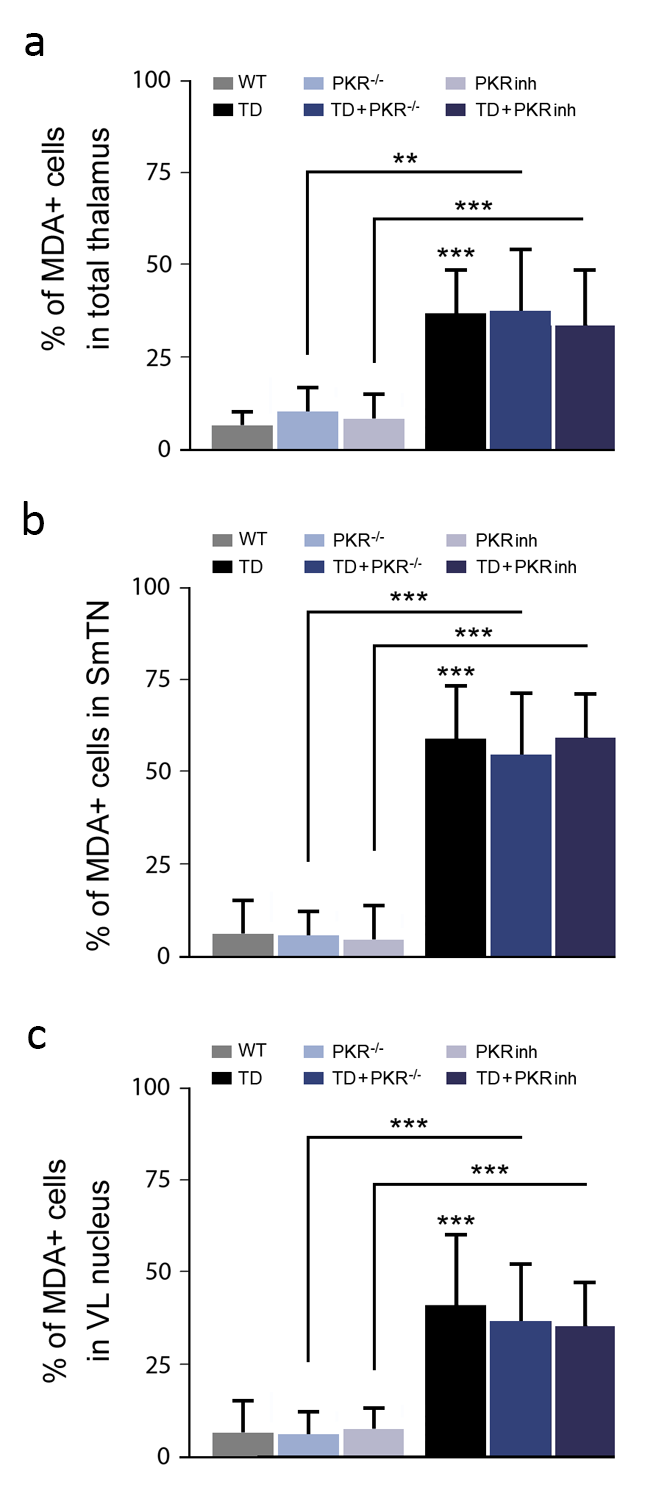


Supplementary Figure 4. Number of MDA (Malondialdehype), a marker of oxidative stress, positive neurons measured on fluorescence mosaic pictures of immunohistochemistry experiment in total thalamus (**a**), and in specific thalamic nuclei SmTN (submedial thalamus nucleus) (**b**) and VL (ventrolateral nucleus) (**c**). Increase of percentage of MDA+ cells due to TD compared to controls did not differ with or without PKR down-regulation. WT [n=8], PKRinh [n=8], PKR^-/-^ [n=7] TD [n=8], TD+PKRinh [n=8] and TD+PKR^-/-^ [n=7].  **p<0.01, ***p<0.001.


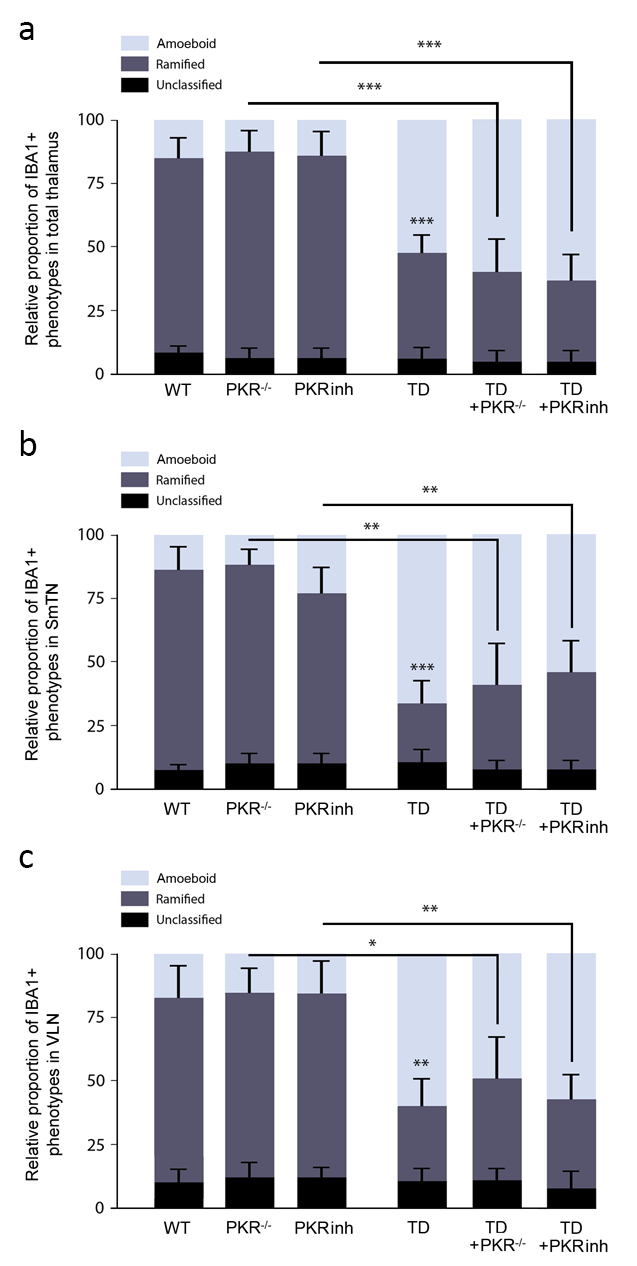


Supplementary Figure 5. Absence of effect of PKR down regulation on thalamic microgial activation. Enhance proportion of IBA1 amoeboid cells still unchanged in TD+PKRinh and TD+PKR^-/-^ mice in total thalamus (**a**), or by considering only SmTN (submedial thalamus) (**b**) and VL (ventrolateral) (**c**) nuclei. WT [n=8], PKRinh [n=8], PKR^-/-^ [n=7] TD [n=8], TD+PKRinh [n=8] and TD+PKR^-/-^ [n=7]. * p<0.05, **p<0.01, ***p<0.001.


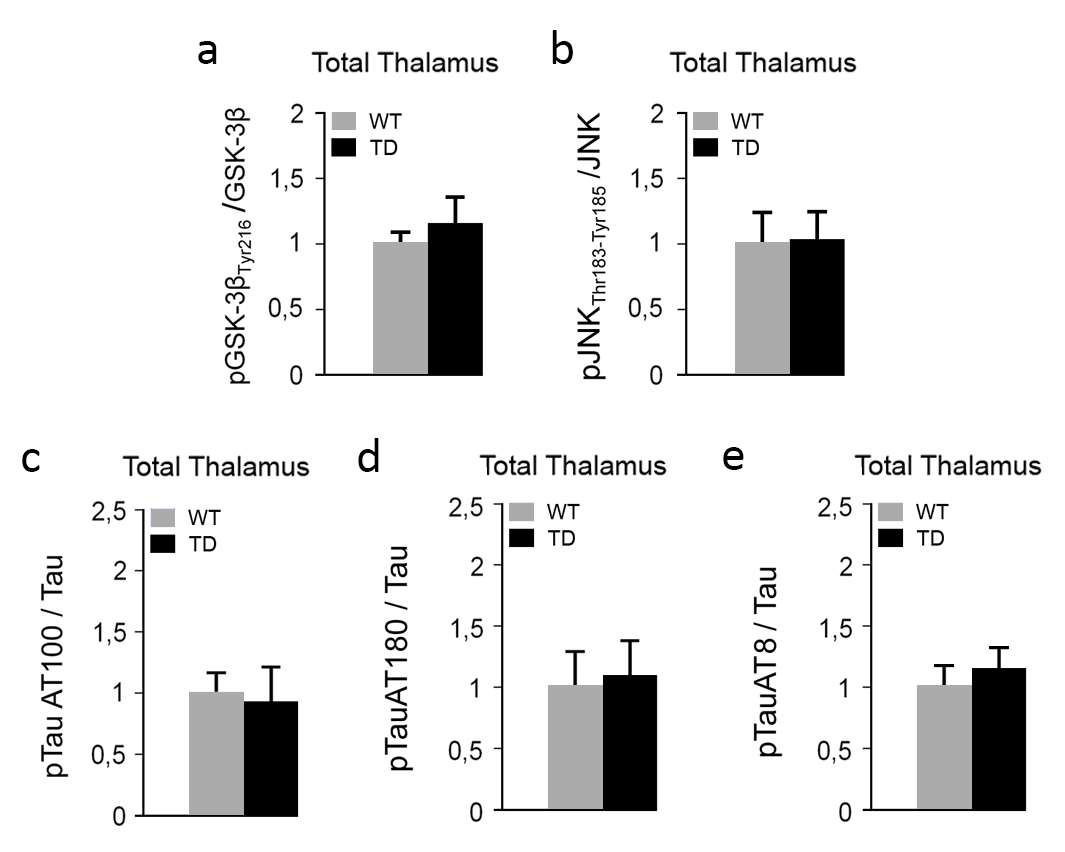


Supplementary Figure 6. Absence of impact of TD diet on the Tau activating pathway. Immunoblot quantification of pGSK-3β_Tyr216_/GSK-3β (**a**), pJNK_Thr183-Tyr185_/JNK (**b**), pTauAT100/Tau (**c**), and pTauAT180/Tau (**d**) ratios did not reveal any variation on their activation degrees. WT [n=12], TD [n=12].


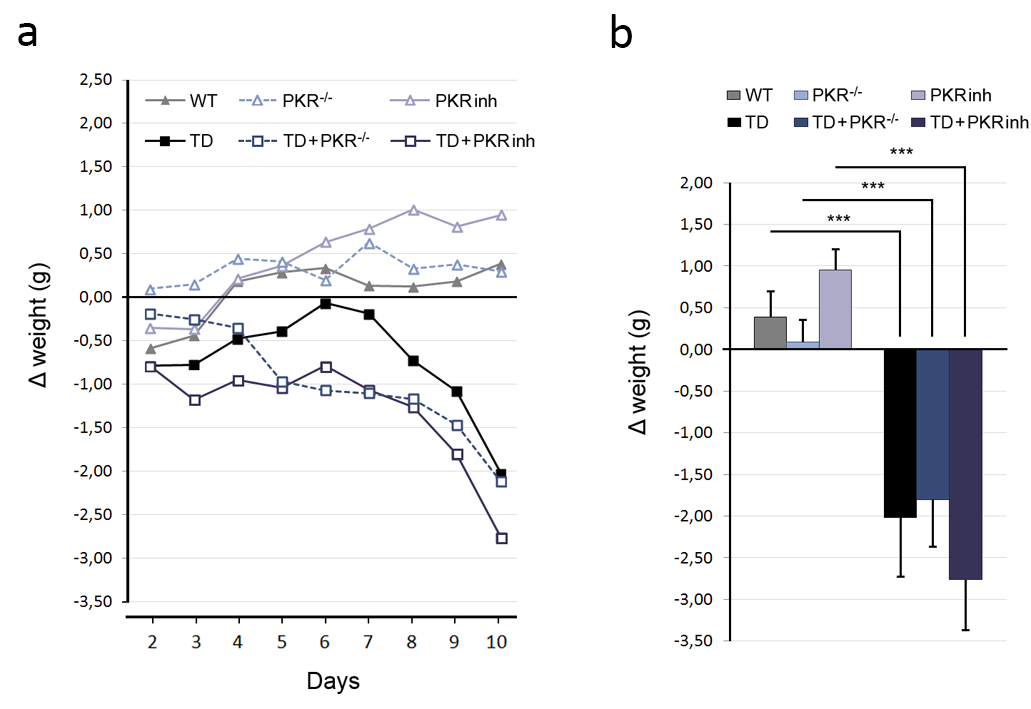


Supplementary Figure 7. Time-course changes in the TD mouse body weight. (**a**) Diagram illustrating body weight evolution at each day of treatment relative to the initial weight measured at day 0. (**b**) Histogram representing the gap between day 0 and day 10 reveals strong weight loss in the 3 groups of TD mice. Data are presented as the mean ± SEM. WT [n=14], PKRinh [n=14], PKR^-/-^ [n=12], TD [n=14], TD+PKRinh [n=14] and TD+PKR^-/-^ [n=12]. ***p<0.001.


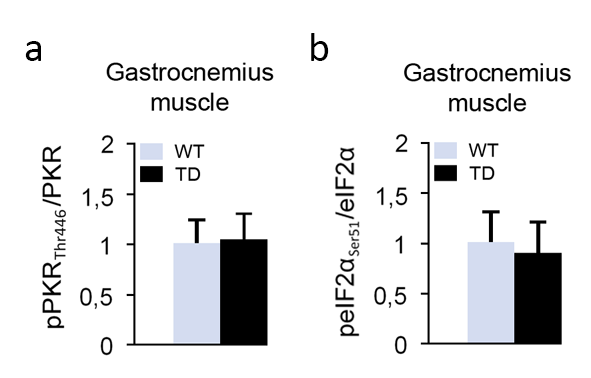


Supplementary Figure 8. Thiamine deficiency in mice did not affect gastrocnemius levels of PKR (**a**) and eIF2α (**b**) activation measured by immunoblot assay. WT [n=8], TD [n=8].
